# Gadd45g Is Essential for Primary Sex Determination, Male Fertility and Testis Development

**DOI:** 10.1371/journal.pone.0058751

**Published:** 2013-03-13

**Authors:** Heiko Johnen, Laura González-Silva, Laura Carramolino, Juana Maria Flores, Miguel Torres, Jesús M. Salvador

**Affiliations:** 1 Department of Immunology and Oncology, Centro Nacional de Biotecnología/Consejo Superior de Investigaciones Científicas, Campus Cantoblanco, Madrid, Spain; 2 Department of Cardiovascular Development and Repair, Centro Nacional de Investigaciones Cardiovasculares, Madrid, Spain; 3 Animal Surgery and Medicine Department, Veterinary School, Universidad Complutense de Madrid, Madrid, Spain; IGBMC/ICS, France

## Abstract

In humans and most mammals, differentiation of the embryonic gonad into ovaries or testes is controlled by the Y-linked gene *SRY*. Here we show a role for the Gadd45g protein in this primary sex differentiation. We characterized mice deficient in Gadd45a, Gadd45b and Gadd45g, as well as double-knockout mice for Gadd45ab, Gadd45ag and Gadd45bg, and found a specific role for Gadd45g in male fertility and testis development. *Gadd45g*-deficient XY mice on a mixed 129/C57BL/6 background showed varying degrees of disorders of sexual development (DSD), ranging from male infertility to an intersex phenotype or complete gonadal dysgenesis (CGD). On a pure C57BL/6 (B6) background, all Gadd45g^−/−^ XY mice were born as completely sex-reversed XY-females, whereas lack of Gadd45a and/or Gadd45b did not affect primary sex determination or testis development. *Gadd45g* expression was similar in female and male embryonic gonads, and peaked around the time of sex differentiation at 11.5 days post-coitum (dpc). The molecular cause of the sex reversal was the failure of Gadd45g^−/−^ XY gonads to achieve the SRY expression threshold necessary for testes differentiation, resulting in ovary and Müllerian duct development. These results identify *Gadd45g* as a candidate gene for male infertility and 46,XY sex reversal in humans.

## Introduction

The Gadd45 (growth arrest and DNA-damage-inducible protein 45) family members Gadd45a, -b and -g are small nuclear and cytoplasmic proteins that bind to and modify the activity of other intracellular proteins, including p21 [1], [2], PCNA [1], [2], CRIF [3], CDK1 [4] and the MAP kinases p38 [5] and MAP3K4 [6]. They are implicated in the regulation of apoptosis, survival, senescence, cell cycle control, DNA repair and the response to physiological or environmental stress in mammalian cells. All family members appear to have overlapping but non-identical functions and binding partners, and are induced by different stimuli [7]. Little is known of their role in embryonic development. It was suggested that loss of an enhancer region that drives brain-specific Gadd45g expression leads to increased growth of brain regions in humans compared to chimpanzees [8]. Another group studied the expression pattern during mouse embryonic development up to 10.5 dpc and proposed a conserved role for Gadd45g in vertebrate neurogenesis [9] as well as involvement in embryonic neural cell development and exit from pluripotency in Xenopus [10].

Here we identify a specific role for Gadd45g in mammalian sex determination. In male gonads, SRY expression triggers differentiation of a somatic support cell lineage into Sertoli cells, which direct the male developmental pathway [11]; in the absence of SRY (in XX gonads), granulosa cells differentiate and the female developmental pathway is activated [12]. These pathways are mutually antagonistic, and disturbances in their molecular network can lead to sex reversal and other disorders of sexual development. We found that Gadd45g, but not Gadd45a or Gadd45b, is necessary for activation of the male sex-determining pathway in mice and its absence leads to development of female gonads. Lack of Gadd45g decreased SRY expression, resulting in ovary and Müllerian duct development, whereas lack of Gadd45a and/or Gadd45b had no effect on testis development.

## Materials and Methods

### Mouse Strains and Genotyping

Gadd45a^−/−^ [13] and Gadd45b^−/−^ mice [14] were generated on a mixed 129/C57BL/6 genetic background. Gadd45g^−/−^ mice were generated by Drs. J.M. Salvador and C. Hollander on a mixed 129/C57BL/6 genetic background (Fig. S1) [15]. Mice were maintained in the CNB animal facility. Gadd45a^−/−^, Gadd45b^−/−^, and Gadd45g^−/−^ mice on a pure C57BL/6 background were generated by backcrossing for seven generations. We mated Gadd45a^−/−^ mice with Gadd45b^−/−^ and Gadd45g^−/−^ mice and intercrossed F1 double heterozygotes to obtain double-null mice. Mouse embryos were obtained by timed mating of Gadd45g^−/−^ or Gadd45g^+/−^ females with Gadd45g^+/−^ males. For embryo staging, midday on the day of vaginal plug appearance was considered 0.5 dpc. Embryos were staged more accurately by counting tail somites posterior to the hind limb bud. Genotyping was done by PCR using the following primers: Gadd45g 5′-GCTGTGCTTTCCGGAACTGTA-3′, 5′-CGGCAGATTTGAGGCT GTGT-3′ and 5′-AGTTGCCAGCCATCTGTTGT-3′; SRY 5′-TCTTAAACTCTGAAGAAG AGAC-3′ and 5′-GTCTTGCCTGTATGTGATGG-3′. Experiments were approved by the CNB Ethical Committee, and mice were handled according to national and European Union animal care standards.

### Isolation of Fetal Gonads from Embryos of Timed-pregnant Mice

Dissection was carried out around noon each day. Pregnant females were sacrificed and embryos extracted. Gonads (including mesonephros) from embryos at different stages were isolated, fixed in 4% paraformaldehyde (1 h) and stored in methanol at −80°C or flash frozen for RNA extraction [16].

### Histological Analysis and Immunohistochemistry

Ovaries and testes from adult mice were fixed in 10% formalin, dehydrated in ascending ethanol concentrations, cleared in xylene and embedded in paraffin wax. Embedded samples were sectioned and hematoxylin/eosin-stained. Gonads were whole-mount stained with anti-SOX9 (sc 20095), -AMH (sc-6886, both from Santa Cruz,) and -PECAM-1-FITC antibodies (Pharmingen). Gonads were rehydrated with TBS, incubated in blocking buffer (1 h), stained with antibodies (overnight, 4°C), mounted with Vectashield Hardset (Source) and analyzed using a Leica TCS SP5 confocal microscope and LAS AF imaging software.

### Quantitative PCR

RNA was extracted from both embryonic gonads (including mesonephros) using the RNeasy Micro Kit (Qiagen). cDNA was synthesized from 100 ng RNA using a High Capacity cDNA Reverse Transcription Kit with RNase Inhibitor (Applied Biosystems). SRY and Gadd45g transcript amounts were quantified using the Taqman gene expression assays Mm00441712_s1 (SRY) and Mm00442225_m1 (Gadd45g)(Applied Biosystems) on an MWG AG BIOTECH Primus 96 thermocycler. All results were normalized to 18S (Fwd: 5′-GAGAAACGGCTACCACATCC-3′; RV: 5′-GGGTCGGGAGTGGGTAAT-3′) housekeeping gene transcripts. In all negative controls (cDNA from female gonads and male no-RT controls), no SRY transcripts were detected.

## Results

### Complete or Partial XY Sex-reversed Phenotype of XY Gadd45g^−/−^ Mice

**Figure 1:**
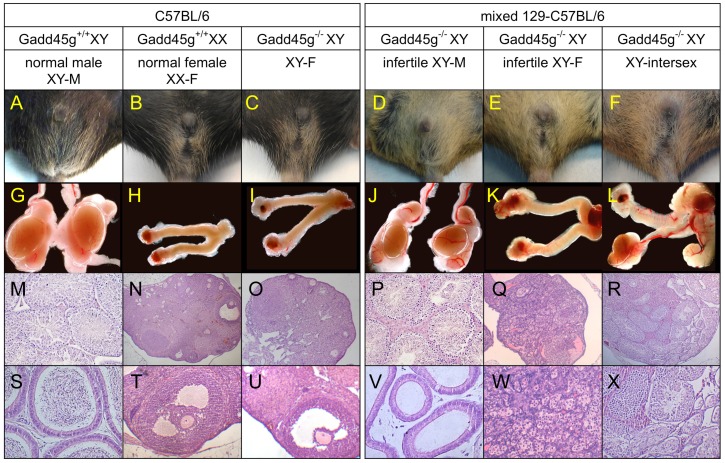
Complete or partial XY sex-reversed phenotype of XY Gadd45g^−/−^ mice on a pure B6 or mixed 129/B6 background. (A–F) External genitals, (G–L) internal reproductive organs (8x magnification), and (M–X) hematoxylin/eosin (HE)-stained gonad or epididymis sections from young adult Gadd45g^−/−^ and wild type mice. External and internal (C,I) reproductive organs from B6 Gadd45g^−/−^ XY female mice (XY-F) are morphologically indistinguishable from wild type females (B, H). HE-stained ovaries from all B6 XY-F mice appear normal and contain oocytes and follicles at different maturation stages (O), including antral follicles with oocytes (U). Corpora lutea are found in XX-F and XY-F ovaries (N,O,T,U). In contrast, a large spectrum of disorders of sexual development (DSD) is seen in Gadd45g^−/−^ XY mice on the 129/B6 background, which can be divided into three groups. 1) Infertile XY-females (E) with female internal reproductive organs (ovary, oviduct, uteri) (K), although the ovaries usually contain only primordial follicles and interstitial cells (Q,W). 2) Infertile XY-males with male phenotype (D) and reproductive system (J; testis, epididymis, vas deferens and seminal vesicles [not shown in J]) with hypoplastic testis (compare J to G). Seminiferous tubules showed reduced spermatogenesis and interstitial cell hyperplasia (P), and no spermatozoa were present in the cauda epididymis (V) and vas deferens. 3) XY-intersex mice (F) with male and female characteristics. External genitals were male, female, or ambiguous (as in F). One side often developed a hypoplastic testis/epididymis/vas deferens (L) and the contralateral gonad, a uterus and a hypoplastic ovary/oviduct/uterus or an ovotestis with mixed ovarian and testicular tissue (L,R,X). Magnification: 5x (N,O,Q,R) or 10x (M,P,S,T,U,V,W,X).

The complete absence of male Gadd45g-deficient mice on the B6 background and the presence of ambiguous genitalia (vagina imperforata, micropenis, abnormal anogenital distance) in some Gadd45g^−/−^ mice on a mixed 129/B6 background prompted us to analyze their genetic sex by PCR amplification of the *SRY* (sex-determining region Y) gene on the Y chromosome. *Gadd45g* deletion did not result in a male-specific lethal phenotype, but the majority of XY Gadd45g^−/−^ mice on the 129/B6 background and all XY Gadd45g^−/−^ mice on the pure B6 background were born as sex-reversed XY females (XY-F) (Fig. 1).

On the B6 background, the external and internal reproductive organs of adult Gadd45g^−/−^ XY-F mice (Fig. 1C,I) were morphologically indistinguishable from those of wild type females (Fig. 1B,H), and histological analysis of the gonads revealed no obvious abnormalities. Hematoxylin/eosin-stained ovaries from all B6 XY-F mice analyzed had oocytes and follicles at different developmental stages (Fig. 1O,U). Corpora lutea (Fig. 1U) were found in all ovaries, indicating that ovulation took place in B6 XY-F mice.

In contrast, the internal and external reproductive organs of 129/B6 Gadd45g^−/−^ XY mice showed a range of DSD that could be broadly categorized in three groups. In the first group, mice had a male phenotype and reproductive system (XY-M), although with testis hypoplasia (Fig. 1D,J). All fertility-tested Gadd45^−/−^ XY-M mice were infertile. Histological analysis showed hypoplastic seminiferous tubules with reduced spermatogenesis and interstitial cell hyperplasia (frequent in infertility; Fig. 1P). No spermatozoa were found in the cauda epididymis and vas deferens (Fig. 1V). Mice in the second group had a female phenotype and reproductive system (XY-F, Fig. 1E,K). These mice had female reproductive organs (ovary, oviduct, uterus), although the ovaries usually contained mainly interstitial cells and multiple primordial follicles, but no other stages of oocyte and follicle development (Fig. 1Q,W). All fertility-tested XY-F mice were infertile. The third group consisted of intersex mice (XY-intersex, Fig. 1F,L) with male, female, or ambiguous external genitals and internal reproductive organs with both female and male characteristics. For example, one side developed a hypoplastic testes/epididymis/vas deferens (Fig. 1L) and the contralateral side, an ovary/oviduct/uterus or an ovotestis with mixed ovarian and testicular tissue in the same gonad (Fig. 1L,R,X).

**Figure 2:**
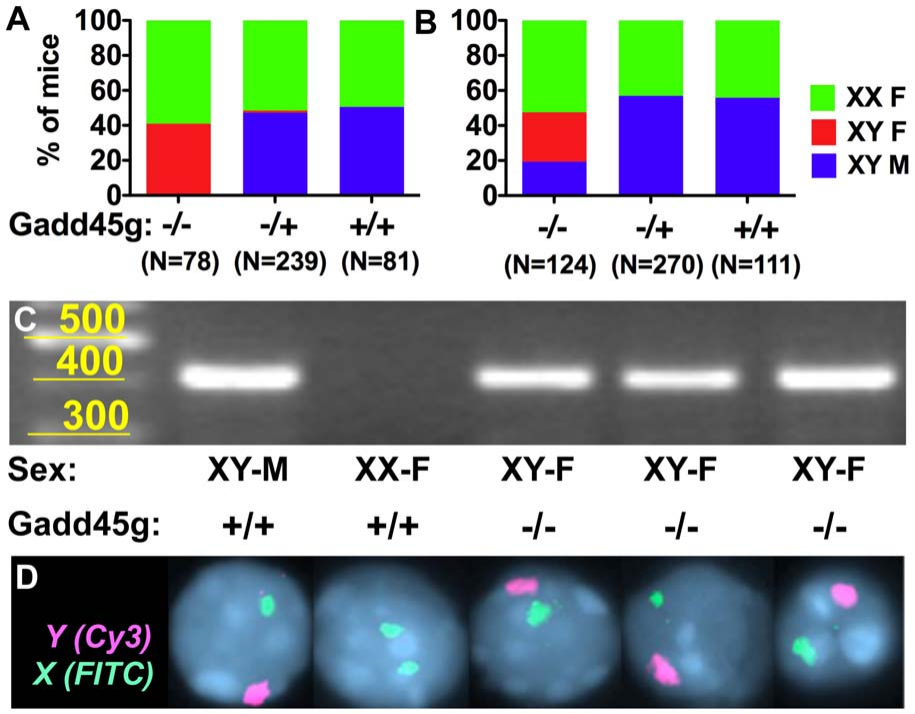
Frequency of sex reversal in distinct Gadd45g genotypes on 129/B6 or B6 backgrounds. (A) *SRY* genotyping showed that 100% of XY Gadd45g^−/−^ and 3% of Gadd45g^+/−^ mice on the B6 background, and (B) 80% of XY Gadd45g^−/−^ mice on the 129/B6 background were born as sex-reversed XY females. (C) Sex reversal in Gadd45g^−/−^ mice born as females was detected by PCR amplification of a 404-bp band from the Y chromosome *SRY* gene. (D) Presence of the Y chromosome in *SRY*-positive Gadd45g^−/−^ XY-F mice was confirmed by chromosome painting (FISH).

The cause for the variability of this phenotype is likely to be the variable contribution of the B6 background in individual mixed-background mice. Compared to the 129 strain, B6 mice are more responsive to disturbances in early testis development [17]. As a result, all B6 Gadd45g^−/−^ XY, and a small number of B6 Gadd45g^+/−^ XY mice showed complete sex reversal (Fig. 2A), whereas some 129/B6 Gadd45g^−/−^ XY mice still developed as (infertile) males (Fig. 2B). Chromosome painting of metaphase X and Y chromosomes confirmed that mice identified as XY-F by PCR (Fig. 2C) carried the Y chromosome (Fig. 2D).

We evaluated the role of Gadd45a and/or Gadd45b in testis development and sex determination. Analysis of >600 single- and double-knockout mice showed that the XY sex reversal is caused by the Gadd45g deletion, independent of the Gadd45a or Gadd45b genotype (Table 1).

**Table 1:** Lack of Gadd45g, but not Gadd45a or Gadd45b leads to male-to-female sex reversal in mice.

		Gadd45g^−/−^				Gadd45g^+/−^				Gadd45g^+/+^			
Gadd45a	Gadd45b	XX	XY*	XX	XY§	XX	XY*	XX	XY§	XX	XY*	XX	XY§
**+/+**	**+/+**	45	24	65	35	79	0	100	0	173	0	100	0
**+/−**	**+/+**	12	7	63	37	13	0	100	0	81	0	100	0
**−/−**	**+/+**	2	1	67	33	4	0	100	0	23	0	100	0
**+/+**	**+/−**	5	2	71	29	19	0	100	0	19	0	100	0
**+/+**	**−/−**	5	5	50	50	6	0	100	0	16	0	100	0
**+/−**	**+/−**	n.a.	n.a.	n.a.	n.a.	n.a.	n.a.	n.a.	n.a.	38	0	100	0
**−/−**	**−/−**	n.a.	n.a.	n.a.	n.a.	n.a.	n.a.	n.a.	n.a.	37	0	100	0
**+/−**	**−/−**	n.a.	n.a.	n.a.	n.a.	n.a.	n.a.	n.a.	n.a.	42	0	100	0
**−/−**	**+/−**	n.a.	n.a.	n.a.	n.a.	n.a.	n.a.	n.a.	n.a.	30	0	100	0
**All genotypes**		69	39	64	36	121	0	100	0	459	0	100	0

* The number of sex-reversed XY females in Gadd45g^−/−^, Gadd45g^+/−^ and Gadd45g^+/+^ mice that also bear a hetero- or homozygous deletion of another Gadd45 family member (Gadd45a or Gadd45b).

§ The percentage of sex-reversed XY females in Gadd45g^−/−^, Gadd45g^+/−^ and Gadd45g^+/+^ mice that also bear a hetero- or homozygous deletion of another Gadd45 family member (Gadd45a or Gadd45b).

n.a., not available.

### Lack of Sertoli Cell Differentiation and Testis Cord Formation in B6 XY Gadd45g^−/−^ Gonads

**Figure 3:**
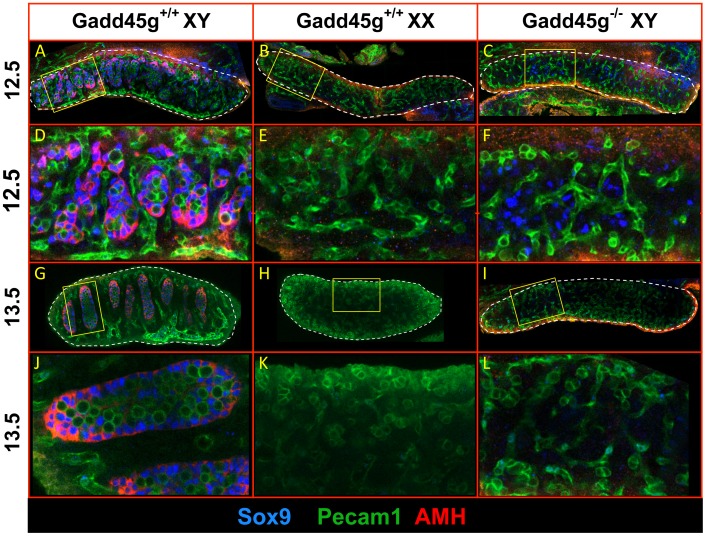
Lack of Sertoli cell differentiation and testis cord formation in XY Gadd45g^−/−^ gonads. (A–L) Confocal optical slices of whole mount immunostained B6 gonads (dashed outline), showing expression of Sertoli cell markers SOX9 (nuclear, blue) and AMH (cytoplasmic, red) and the germ/endothelial cell marker Pecam1 (membrane, green). (A) From 12.5 dpc, wild type male gonads can be distinguished morphologically from female gonads (B) by the appearance of testis cord structures containing SOX9-expressing Sertoli cells. (C) In Gadd45g^−/−^ XY gonads, only transient SOX9 expression was observed in a small number of somatic cells at 12.5 dpc. (D–F) and (J–L) are enlarged details of the images above. (G) At 13.5 dpc, all germ cells in male gonads are enclosed in testis cords and interact closely with a surrounding single layer of SOX9- and AMH-positive Sertoli cells. (H,I,K,L) No testis cord formation or AMH expression was induced in Gadd45g^+/+^ XX (H,K) or in Gadd45g^−/−^ XY (I,L) gonads.

We tested whether lack of Gadd45g affects key molecular pathways during the time window for gonadal sex differentiation in mice [18], [19]. At day 10 dpc, the gonads arise from the genital ridge on the surface of the mesonephros. At this stage, they contain bipotential somatic and germ cells that can follow the male or female pathway. In XY gonads, *SRY* expression by a somatic supporting cell lineage (pre-Sertoli) induces *SOX9* expression (Fig. 3A,D,G,J), resulting in Sertoli cell differentiation and testis development. Starting at 13.5 dpc, Sertoli cell-derived AMH (anti-Müllerian hormone) (Fig. 3G,J) inhibits development of the Müllerian ducts in the male embryo, and testes-derived testosterone directs development of the adjacent Wolffian duct into epididymis, vas deferens and seminal vesicles. In the absence of SRY in XX gonads, *SOX9* is downregulated (Fig. 3B,E,H,K) and a female-specific gene expression program is activated, leading to differentiation of the somatic supporting lineage into granulosa cells, which support oocyte development [18]. We observed a striking decrease in the number of SOX9-positive somatic cells in Gadd45g^−/−^ XY gonads (Fig. 3C,F) compared to wild type controls (Fig. 3A,D). Testis cord formation and AMH expression were not observed (Fig. 3I,L) and SOX9 was not detected after 12.5 dpc. The results indicated that Gadd45g exerts its effects on or before 12.5 dpc. Reduction of SOX9 expression causes primary male-to-female sex reversal in mice [20] and humans [21].

### Gadd45g is Necessary for *SRY* Expression and Testis Development at the Time of Primary Sex Determination

**Figure 4:**
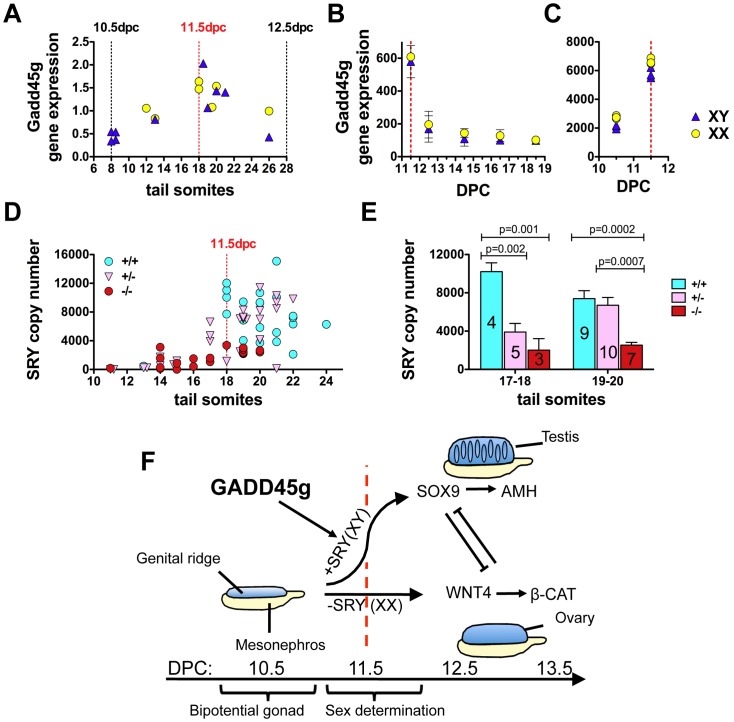
Gadd45g is necessary for *SRY* expression and testis development at the time of primary sex determination of the bipotential embryonic gonad. (A) RT-PCR quantification of relative *Gadd45g* expression in B6 wild type XX and XY embryonic gonads (including mesonephros), collected between 10.5 (8 tail somites) and 12.5 dpc (28 tail somites). In all images, the dashed red line denotes the point of maximal *SRY* expression (11.5 dpc or 18 tail somites). (B) Microarray quantification of relative *Gadd45g* expression in normal wild type XX and XY embryonic gonads (including mesonephros) from the time of the bipotential gonad (GEO data set GSE6916). (C) Microarray quantification of relative *Gadd45g* expression in purified somatic support precursor cells during the critical sex determination period (10.5–11.5 dpc) (GEO data set GDS1724). (D) RT-PCR quantification of *SRY* copy numbers in Gadd45g^+/+^, Gadd45g^+/−^ and Gadd45g^−/−^ gonads (including mesonephros) between 10.5–12.5 dpc. (E) RT-PCR quantification (mean and SEM) of SRY copy numbers in Gadd45g^+/+^, Gadd45g^+/−^ and Gadd45g^−/−^ gonads (including mesonephros) at the 17 to 21 tail somite stage. Statistical analysis (Student’s t-test) of all data from Fig. 4D at the 17–18 or 19–20 tail somite stage showed reduced SRY expression in Gadd45g^−/−^ mice and delayed SRY upregulation in Gadd45g^+/−^ mice compared to wild type controls. Numbers for each group (*n*) are displayed in each column. (F) Main components of the transcriptional network around the time of primary sex determination and suggested mechanism of Gadd45g action. In the absence of *SRY* (XX gonads), female-specific genetic programs (such as the WNT4/beta-catenin pathway) direct differentiation of the somatic support lineage into granulosa cells and ovary development. In XY gonads, *SRY* expression at 11.5 dpc induces *SOX9* expression, Sertoli cell differentiation and testis cord formation. In males, Sertoli cell-derived anti-Müllerian hormone (AMH) induces Müllerian duct regression, and testosterone induces differentiation of the Wolffian duct into vas deferens, seminal vesicles and epididymis. AMH acts ipsilaterally, which explains the intersex phenotype shown in Fig. 1L. In Gadd45g-deficient mice, SRY expression in XY gonads fails to reach the threshold level necessary for testis differentiation. The dashed red line denotes the point of maximal SRY expression (11.5 dpc).

We analyzed Gadd45g expression in male and female gonads during this period by quantitative PCR and found that, unlike many other molecules important for sex determination, the Gadd45g expression pattern was not sexually dimorphic. *Gadd45g* levels were similar in wild type XY and XX gonads during the sex determination period, and peaked at the time of primary sex differentiation (11.5 dpc, or 18 tail somites), when *SRY* is also expressed (Fig. 4A). Analysis of *Gadd45g* expression using microarray gene expression data downloaded from GEO (http://www.ncbi.nlm.nih.gov/geo) also showed that *Gadd45g* was detected at similar levels in both female and male gonads on day 11.5 dpc and declined thereafter (Fig. 4B) [22]. Data from another study using purified Sertoli/granulosa precursor cells (Fig. 4C) also showed similar *Gadd45g* expression in both sexes, and maximum levels at 11.5 dpc [23]. Microarray quantification of relative Gadd45a and Gadd45b levels showed lack of Gadd45a and Gadd45b expression in purified somatic supporting precursor cells (Fig. S2). Only Gadd45g expression was induced robustly in embryonic gonads and in somatic precursor cells at 11.5 dpc (Fig. S2B).

We used quantitative PCR to quantify *SRY* expression levels in B6 Gadd45g^−/−^ XY gonads compared to wild type XY controls, and found that the molecular cause of sex reversal was the failure of Gadd45g^−/−^ XY gonads to upregulate *SRY* (Fig. 4D). Gadd45g^+/−^ XY gonads showed a reduction in *SRY* expression at the 17–18 tail somite stage compared to wild type controls (Fig. 4E), although wild type *SRY* levels were detected thereafter. As a consequence, gonad development in all B6 Gadd45g^−/−^ XY mice and 3% of Gadd45g^+/−^ XY mice followed the female developmental pathway (Fig. 2A, Fig. 4F).

## Discussion

Our study shows that of the three members of the Gadd45 family, Gadd45g, but not Gadd45a or Gadd45b is essential in testis development, male fertility and sex determination. We characterized mice deficient in each of the Gadd45 isoforms, as well as double-knockout mice for Gadd45ab, Gadd45bg and Gadd45ag, and found a specific role for Gadd45g in sex determination. Lack of Gadd45g decreased SRY expression and blocked SOX9 expression, resulting in ovary and Müllerian duct development. The data indicate that Gadd45g is the only Gadd45 family member that regulates SRY expression. Our results also emphasize the relevance of genetic background in mouse models with sex reversal phenotype. We found a more pronounced phenotype in Gadd45g^−/−^ mice generated on the B6 than on the 129SvJ-C57/B6 background. Although other groups have developed Gadd45g^−/−^ mice, it is possible that sex reversal was not observed because most experiments used mutants on a pure 129SvJ or mixed 129SvJ-C57/B6 backgrounds.

Compared to the testis-determining pathways downstream of *SRY* in male and the ovary-determining pathways in female gonads, little is known of the pathways that regulate *SRY* itself (for a review, see [12]). The insulin receptor tyrosine kinase gene family (IGF1R, IR, IRR) [24], the transcription factor GATA4 and its co-factor FOG2 [25], the +KTS isoform of the WT1 transcription factor [26], as well as MAP3K4 [27] upregulate *SRY* expression. Many known biological functions of Gadd45g are mediated by its ability to bind and activate MAP3K4, a mitogen-activated protein kinase kinase kinase (MAPKKK). Bogani *et al.* reported male-to-female sex reversal in MAP3K4-deficient mice caused by decreased SRY expression, although embryos die pre- or perinatally due to unrelated developmental defects [27]. Sex reversal was recently associated with reduced phosphorylation of p38 MAPK and GATA4, suggesting that Gadd45g is needed to promote MAP3K4-mediated activation of p38 signaling in murine embryonic gonadal somatic cells for testis determination [28]; [29]. It remains to be determined whether these factors alter SRY levels in individual cells or the proliferation of SRY-expressing cells, and how MAPK activation leads to increased *SRY* transcription.

Deletion of many of the genes that regulate *SRY* expression lead to embryonic or perinatal death in mice (e.g., GATA4, FOG2, or MAP3K4), or are accompanied by additional developmental complications in humans (for example, Frasier syndrome in patients with a mutant WT1 isoform); nonetheless, we detected no developmental defects other than sex reversal or infertility in Gadd45g^−/−^ mice. In humans, abnormal appearance of genital organs occurs in one of every 4500 births [30], and it is estimated that as many as 1.7% of live births show some degree of DSD [31]; infertility affects 10–15% of couples, with similar contributions from both sexes [32]. The genetic basis of human male-to-female sex reversal remains unexplained in the majority of cases. As there are no known descriptions of human Gadd45g^−/−^ individuals, it is currently not possible to determine whether the effect is the same or similar in man. The complete sex reversal phenotype in Gadd45g^−/−^ mice and the fact that Gadd45g is a key upstream activator of the master regulator SRY nonetheless suggest that it is a promising candidate gene in human non-syndromic male infertility and in partial or complete male-to-female primary sex reversal in 46, XY individuals.

## Supporting Information

Figure S1 **Generation of Gadd45g^−/−^ mice.** (A) Targeting strategy. The endogenous Gadd45g contains 4 exons. A fragment of Gadd45g including exons 1–3 was replaced with a PMG-neo cassette. A XbaI site introduced in the PMG-neo and one genomic XbaI site external to the targeting construct were used for determination of homologous recombination by Southern blot analysis, using a flanking probe (first line). (B) Southern blot analysis of target ES cells clones yielded a ∼2.9 Kb hybridizing fragment corresponding to endogenous Gadd45g, whereas the disrupted allele was 3.7 Kb. (C) PCR genotyping of tail DNA from Gadd45g^+/+^, Gadd45g^+/−^ and Gadd45g^−/−^ mice. To detect the wild type allele, we used the primers JS1 (5′-GCTGTGCTTTCCGGAACTGTA-3′) and JS2 (5′-CGGCAGATTTGAGGC TGTGT-3′), which generated a 335 bp band. The deleted allele was detected using JS2 and JS4 (5′-AGTTGCCAGCCATCTGTTGT-3′), which produced a 486 bp product.
(TIF)

Figure S2 **Lack of Gadd45a and Gadd45b expression in purified somatic supporting precursor cells.** (A) Microarray quantification of relative Gadd45a, Gadd45b and Gadd45g expression in wild type XX and XY embryonic gonads (including mesonephros) from the time of the bipotential gonad (GEO data set GSE6916). (B) Microarray quantification of relative Gadd45a, Gadd45b and Gadd45g expression in purified somatic supporting precursor cells during the critical sex determination period (10.5–11.5 dpc) (GEO data set GDS1724).
(TIFF)
