# Supplementary figures and images for: Gadd45g Is Essential for Primary Sex Determination, Male Fertility and Testis Development
Source: PLoS One. 2013 Mar 13;8(3):e58751. doi: 10.1371/journal.pone.0058751 (PMC3596291; doi:10.1371/journal.pone.0058751)

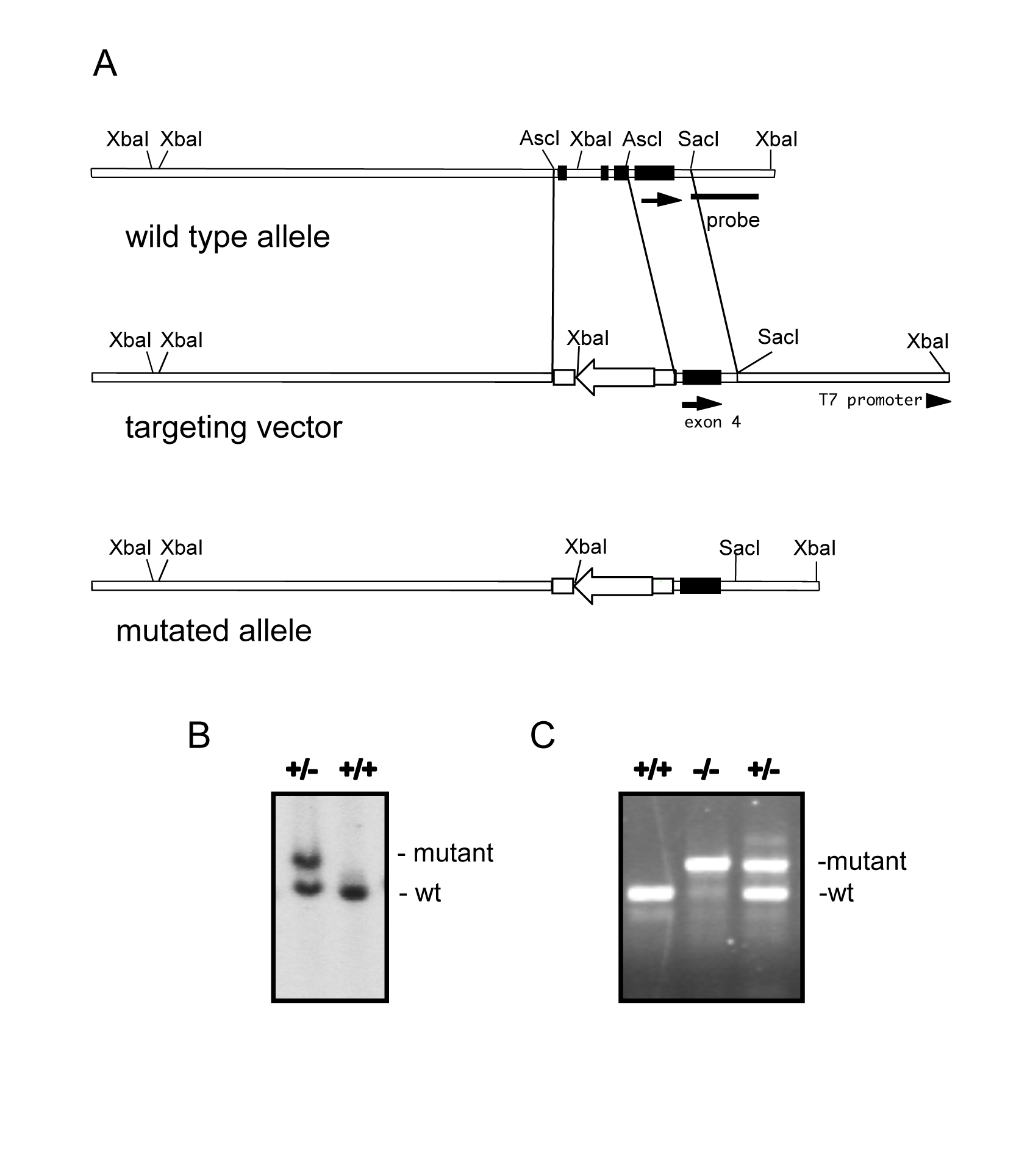

Supplement: Figure S1 — Generation of Gadd45g−/− mice. (A) Targeting strategy. The endogenous Gadd45g contains 4 exons. A fragment of Gadd45g including exons 1–3 was replaced with a PMG-neo cassette. A XbaI site introduced in the PMG-neo and one genomic XbaI site external to the targeting construct were used for determination of homologous recombination by Southern blot analysis, using a flanking probe (first line). (B) Southern blot analysis of target ES cells clones yielded a ∼2.9 Kb hybridizing fragment corresponding to endogenous Gadd45g, whereas the disrupted allele was 3.7 Kb. (C) PCR genotyping of tail DNA from Gadd45g+/+, Gadd45g+/− and Gadd45g−/− mice. To detect the wild type allele, we used the primers JS1 (5′-GCTGTGCTTTCCGGAACTGTA-3′) and JS2 (5′-CGGCAGATTTGAGGC TGTGT-3′), which generated a 335 bp band. The deleted allele was detected using JS2 and JS4 (5′-AGTTGCCAGCCATCTGTTGT-3′), which produced a 486 bp product. (TIF) [file pone.0058751.s001.tif]

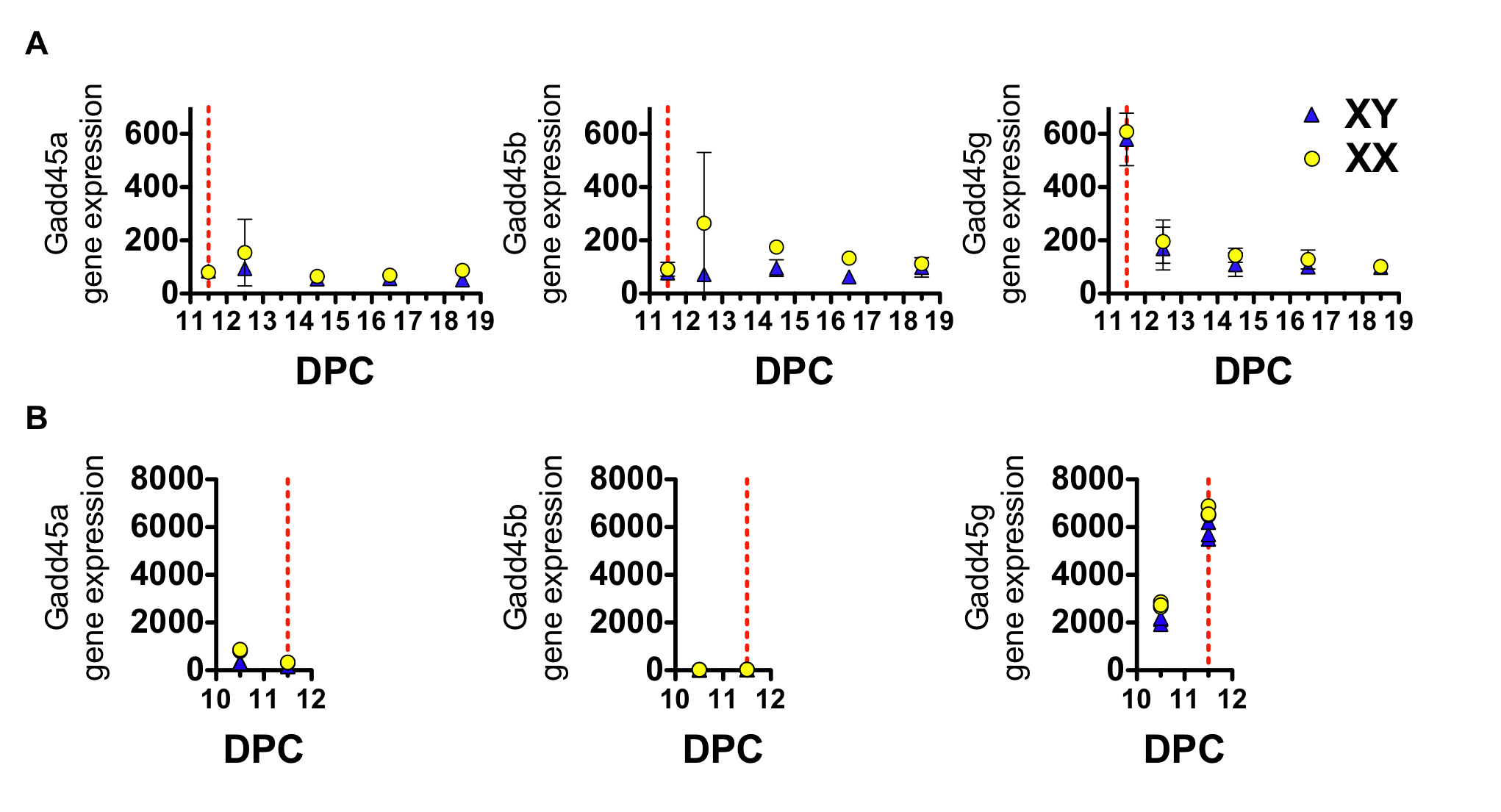

Supplement: Figure S2 — Lack of Gadd45a and Gadd45b expression in purified somatic supporting precursor cells. (A) Microarray quantification of relative Gadd45a, Gadd45b and Gadd45g expression in wild type XX and XY embryonic gonads (including mesonephros) from the time of the bipotential gonad (GEO data set GSE6916). (B) Microarray quantification of relative Gadd45a, Gadd45b and Gadd45g expression in purified somatic supporting precursor cells during the critical sex determination period (10.5–11.5 dpc) (GEO data set GDS1724). (TIFF) [file pone.0058751.s002.tiff]
